# Genome-wide association mapping of Fusarium crown rot resistance in *Aegilops tauschii*

**DOI:** 10.3389/fpls.2022.998622

**Published:** 2022-09-30

**Authors:** Yu Lin, Qing Wang, Hao Chen, Ning Yan, Fangkun Wu, Zhiqiang Wang, Caixia Li, Yaxi Liu

**Affiliations:** ^1^State Key Laboratory of Crop Gene Exploration and Utilization in Southwest China, Chengdu, China; ^2^Triticeae Research Institute, Sichuan Agricultural University, Chengdu, China

**Keywords:** *Aegilops tauschii* ssp. *tauschii*, *Aegilops tauschii* ssp. *strangulata*, candidate gene, FCR resistance, GWAS, QTL, resistance allele, SNP

## Abstract

Fusarium crown rot (FCR), caused by various *Fusarium* species, is a primary fungal disease in most wheat-growing regions worldwide. *A. tauschii*, the diploid wild progenitor of the D-genome of common wheat, is a reservoir of genetic diversity for improving bread wheat biotic and abiotic resistance/tolerance. A worldwide collection of 286 *A. tauschii* accessions was used to evaluate FCR resistance. Population structure analysis revealed that 115 belonged to the *A. tauschii* ssp. *strangulata* subspecies, and 171 belonged to the *A. tauschii* ssp. *tauschii* subspecies. Five accessions with disease index values lower than 20 showed moderate resistance to FCR. These five originated from Afghanistan, China, Iran, Uzbekistan, and Turkey, all belonging to the *tauschii* subspecies. Genome-wide association mapping using 6,739 single nucleotide polymorphisms (SNPs) revealed that two SNPs on chromosome 2D and four SNPs on chromosome 7D were significantly associated with FCR resistance. Almost all FCR resistance alleles were presented in accessions from the *tauschii* subspecies, and only 4, 11, and 19 resistance alleles were presented in accessions from the *strangulata* subspecies. Combining phenotypic correlation analysis and genome-wide association mapping confirmed that FCR resistance loci were independent of flowering time, heading date, and plant height in this association panel. Six genes encoding disease resistance-related proteins were selected as candidates for further validation. The identified resistant *A. tauschii* accessions will provide robust resistance gene sources for breeding FCR-resistant cultivars. The associated loci/genes will accelerate and improve FCR in breeding programs by deploying marker-assisted selection.

## Introduction

Fusarium crown rot (FCR), caused by various *Fusarium* species, is a severe fungal disease that affects the whole growth period of the plant in cereal crops, such as wheat, barley, sorghum, and so on (Li et al., 2012; Xu et al., 2017; Cuevas et al., 2019). It poses a considerable threat to wheat and barley production in regions with arid and semi-arid cropping globally, including major wheat-producing countries, such as Australia, the USA, Canada, New Zealand, China, and many others (Kazan and Gardiner, 2018). For example, yield loss estimates have indicated that FCR can cause a 35% reduction in wheat grain yield under natural inoculum levels in the Pacific Northwest of the USA (Smiley et al., 2005). Besides, FCR is estimated to routinely cause a 10% yield loss in cereals in Australia (Murray and Brennan, 2009). In China, FCR has become one of the major diseases that have caused wheat and barley yield losses in the past decade. It has generated more than 70% yield losses at individual sites with an average annual reduction of 9–35% from 2008 to 2019 (Li et al., 2012; Xu et al., 2016, 2017; Luan et al., 2022). In addition, cereal crop grains infected by *Fusarium* species will accumulate mycotoxins, which are harmful to humans and livestock. Developing resistant cereal crop cultivars is the most practical and sustainable to control FCR.

Marker-assisted selection is an important approach for breeding, which can improve the efficiency of breeding. FCR resistance genes/loci have been identified in chromosomes 1A, 1B, 1D, 2A, 2B, 2D, 3A, 3B, 3D, 4B, 4D, 5D, 6A, 6B, and 6D in wheat (Liu C. et al., 2015; Su et al., 2021; Yang et al., 2021) and 1H, 2H, 3H, 4H, 5H, and 6H in Barley (Chen et al., 2013; Liu C. et al., 2015; Gao et al., 2019). In wheat, 12 single nucleotide polymorphisms (SNPs) within a 0.5-Mb genomic region were identified by a genome-wide association study (GWAS) using the Wheat 660 K SNP array in an association panel containing 435 introgression lines (Yang et al., 2021). A candidate gene, *TraesCS4B02G385500* (also known as *TaDIR-B1*: encoding dirigent protein), was cloned and confirmed to improve resistance FCR resistance by virus-induced gene silencing and EMS-mutagenized wheat lines (Yang et al., 2021). Using a set of 358 Chinese germplasms, a novel 13.78 Mb region targeted by five SNPs on chromosome arm 5DL was identified by GWAS using the wheat 55 K SNP array (Jin et al., 2020). Several candidate genes within this 5DL region encode TIR-NBS-LRR proteins associated with FCR resistance (Jin et al., 2020). Although these studies provided some important markers associated with FCR resistance, more major and stable genetic loci controlling the resistance to FCR are needed to deal with this severe fungal disease in wheat.

*Aegilops tauschii* (2*n* = 2 × = 14, DD) is the D-genome progenitor of common wheat. The diverse *A. tauschii* D-genome offers a valuable gene pool for biotic and abiotic resistance/tolerance, such as phosphorus-deficient tolerance (Liu et al., 2015a), drought resistance (Qin et al., 2016), stripe rust resistance (Zhang et al., 2019b), powdery mildew resistance (Xue et al., 2022), and so on. Although the hexaploid *Triticum aestivum* (AABBDD) was formed by hybridization with *Triticum turgidum* (AABB) and *A. tauschii* (DD), only a tiny fraction of the D- genome subpopulation participated in the hybridization (Giles and Brown, 2006; Zhou et al., 2020). Harnessing genetic diversity from wild wheat progenitor *A. tauschii* to improve hexaploid wheat has been proven fast and efficient (Zhang et al., 2019b; Awan et al., 2022; Gaurav et al., 2022). Here, using 286 *A. tauschii* accessions, we aim to (1) identify FCR resistant materials in *A. tauschii*; (2) identify loci significantly associated with FCR resistance using GWAS based on SNP array; (3) select candidate genes for loci of FCR resistance.

## Materials and methods

### Plant materials

A total of 286 *A. tauschii* accessions were used in the present study. Based on the morphological classification, 115 and 171 belong to *A. tauschii* ssp. *strangulata* and *A. tauschii* ssp. *tauschii*, respectively (Supplementary Table 1).

### Filed experiment design and phenotypic evaluation

All accessions were planted for three straight years during the sowing seasons in October from 2017 to 2019. Five seeds of each accession were planted in three rows as previously described (Liu et al., 2015b; Wang et al., 2021). The row length was 1.5 m, and the space between rows was 0.6 m. The heading date and flowering time were recorded for each accession in Wenjiang from 2018 to 2020 and in Chongzhou in 2020. Five plants of each accession were used to evaluate plant height. At physiological maturity in 2020, spikes of each accession were harvested, and hand-threshed seeds were used to identify FCR resistance further.

### Identification of Fusarium crown rot resistance

A highly aggressive *Fusarium pseudograminearum* isolate (Fp.322) was used in FCR inoculation. The inoculum preparation, injection, and FCR resistance identification were based on the method described by a previous study (Li et al., 2008) with some modifications. Briefly, the Fp.322 was incubated on half-strength potato dextrose agar plates at 25°C for 7 days. When the white mycelium grew over the leaves and showed peach-red to dark-red pigmentation, a piece of culture medium with mycelium was taken out from the margin of the plate and then transferred into a conical flask containing 100 ml carboxy-methyl-cellulose sodium liquid medium. The conical flask was put into a shaking incubator at 180 rpm at 28°C for approximately 6 days. The concentration of spore suspension was adjusted to 1 × 10^6^ spores per ml. The Tween-20 was added to the spore suspension to a final concentration of 0.1% volume ratio before use for injection.

The seedling disease assessment contained three replicates. Twenty unified seeds of each accession were used for each replicate. Seeds were washed with 10% NaClO for 20 min and with sterile water three times. Seeds were soaked in water at 45°C for 5 min to break dormancy. Then, seeds were put in Petri dishes on three layers of filter paper saturated with water and germinated at 25°C in an incubator. Ten seedlings with the shoot length at 0.5–1.0 cm for each accession were immersed in the spore suspension for 1 min and were sown in 5-cm square seeding trays containing autoclaved potting mix. The seedlings were grown in the greenhouse at Sichuan Agricultural University with a 16 h photoperiod at 25/22 (±1) °C day/night temperature and 65/85% day/night relative humidity. Seedlings were watered only when wilt symptoms promoted FCR development, as in a previous study (Jin et al., 2020).

When the whole plants of “Janz” were severe to completely necrotic, the FCR severity of each accession was recorded using a 0–5 scale (Li et al., 2008). The FCR resistance was identified using the disease index (DI). The DI value of each replication was calculated according to the formula:

DI = (∑*nX*/5*N) × 100*

where *X* is the scale value of each plant, *n* is the number of plants in the category, and *N* is the total number of plants assessed for each line (Jin et al., 2020). The DI value represented FCR resistance was used for further analysis.

### Phenotypic statistical analysis

The mean value, minima, maxima, coefficient of variation (CV), and Person’s correlation coefficient (*r*) were calculated using IBM SPSS Statistics 20 (IBM Corp., Armonk, NY, USA). The analysis of variance was calculated using the “GLM” procedure in SAS 9.4 (SAS Institute Inc., Cary, NC, USA). The broad-sense heritability was estimated using the formula:

*H*^2^ = *Vg*/(*Vg* + *Vge* + *Ve*)

where *Vg*, *Vge*, and *Ve* are the estimates of genetic variance, the genotype × environment interaction, and environmental variance, respectively (Smith et al., 1998). The best linear unbiased prediction (BLUP) values of each trait were calculated in SAS 9.4 (SAS Institute Inc., Cary, NC, USA) to reduce environmental effects.

### Genotyping, population structure, and kinship analysis

DNA samples of each genotype were extracted from young leaves using the CTAB method (Murray and Thompson, 1980). All 286 accessions were genotyped using the Illumina 10 K SNP arrays. The physical location of SNPs was based on *A. tauschii* reference genome v4.0 (Aet v4.0) (Luo et al., 2017). The gathered SNPs with minor allele frequency (MAF) <0.05, heterozygosis >0.20, or missing data >0.20 were rejected for further analysis. Finally, 6,739 SNPs were used for population structure analysis STRUCTURE 2.3.4 based on the linkage ancestry model (Pritchard et al., 2000). The *K* value was set from 1 to 10, with five runs per *K*. A total of 10,000 burn-in iterations and 10,000 Markov Chain Monte Carlo iterations were developed for each *K*. The best *K* was determined using the Evanno method (Evanno et al., 2005) calculated by web-based STRUCTURE HARVESTER (Earl and VonHoldt, 2012). The population structure matrix was gathered using the CLUMPP among five repetitions (Jakobsson and Rosenberg, 2007). A neighbor-joining tree was created using TASSEL 5.0 (Bradbury et al., 2007) and visualized using the iTOL website (Letunic and Bork, 2021).

### Genome-wide association analysis and candidate gene prediction

The mixed linear model adjusted by population structure and kinship was used to identify marker-trait associations in TASSEL 5.0 (Yu et al., 2006; Bradbury et al., 2007). The significance threshold was set at *P* < 0.001 correspondingly −log_10_(*P*) = 3.00. Manhattan plots of GWAS results were plotted in R 3.6.3 (R Core Team, 2014) using the package “CMplot.” Flanking sequences of significantly associated SNPs and reported QTL were used to obtain chromosomal information and physical distances from Chinese Spring Reference Sequence v2.1 (IWGSC RefSeq V2.1) (Zhu et al., 2021). The physical map of IWGSC RefSeq V2.1 was used as the reference genome to determine whether significantly associated loci in *A. tauschii* and significant QTL in the D-genome of wheat were overlapped.

According to previously reported linkage disequilibrium decay distances in *A. tauschii* (Wang et al., 2021; Gaurav et al., 2022), conservative genomic regions (500 kb upstream and downstream of the significant SNPs) were used to select candidate genes. The predicted high confidence (HC) genes in these genomic regions of reference genome Aet v4.0 were selected to annotate using arabidopsis as background species by KOBAS 3.0 (Bu et al., 2021). Candidate genes were identified based on annotated function information. Candidate genes were identified based on annotated function information.

## Results

### Single nucleotide polymorphisms distribution and population structure of the *Aegilops tauschii* natural population

A total of 6,739 high polymorphic SNPs (MAF ≥0.05, both missing and heterozygous ≤0.20) were gathered for further analysis (Supplementary Figure 1 and Table 1). These SNPs are evenly distributed over seven chromosomes corresponding to a total map length of 4,022.29 Mb. The number of SNPs ranged from 707 for chromosome 6D to 1,237 for chromosome 2D. For marker density, the average distance between two SNPs ranged from 0.53 Mb on chromosome 2D to 0.70 Mb on chromosome 6D, with an average value of 0.60 for all chromosomes. The average polymorphism information content was 0.43 for 6,739 SNPs showing a polymorphism.

**TABLE 1:** Distributions of single nucleotide polymorphisms, density, and polymorphism information content of 286 *Aegilops tauschii* accessions.

Chromosome	Number of marker	Physical map length (Mb)	Marker density (Mb/SNP)	Polymorphism information content
1D	901	502.18	0.56	0.42
2D	1,237	651.08	0.53	0.43
3D	1,029	626.33	0.61	0.43
4D	785	525.89	0.67	0.45
5D	974	576.73	0.59	0.42
6D	707	495.52	0.70	0.42
7D	1,106	644.57	0.58	0.43
ALL	6,739	4,022.29	0.60	0.43

**FIGURE 1:**
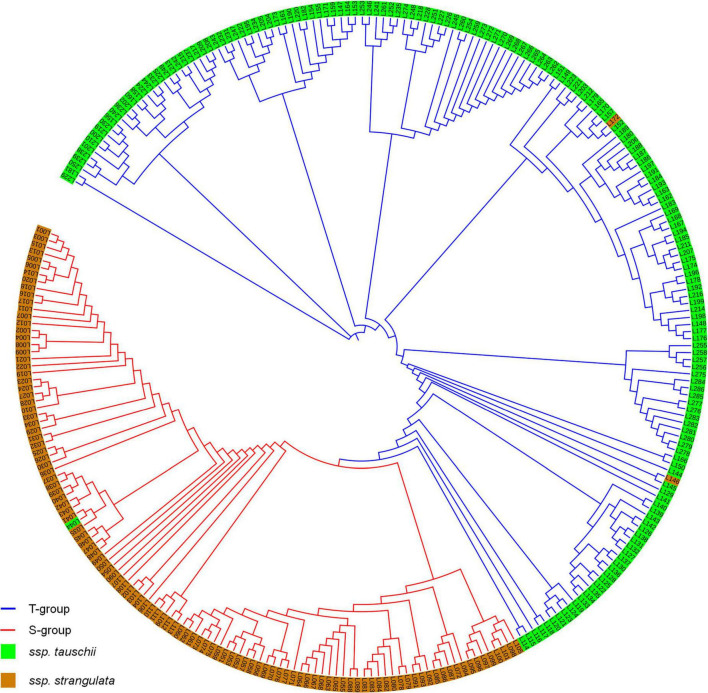
Population structures of 286 *Aegilops tauschii* revealed by STRUCTURE 2.3.4 and neighbor-joining tree.

Population structure analysis showed that Delta *K* had the highest value while *K* = 2 (Supplementary Figure 2). Thus, 286 accessions were divided into two groups. The first group included 114 *A. tauschii* ssp. *strangulata* accessions and one *A. tauschii* ssp. *tauschii* accessions, and the second group had 169 *A. tauschii* ssp. *tauschii* accessions and two *A. tauschii* ssp. *strangulata* accessions. Almost all *strangulata* accessions were divided into the first group (S-group), and *tauschii* accessions were divided into the second group (T-group). The cluster analysis by neighbor-joining tree also divided 286 accessions into two groups confirming the result revealed by population structure (Figure 1).

### Genetic variation of Fusarium crown rot resistance in *Aegilops tauschii* and correlations with flowering time, heading date, and plant height

**FIGURE 2:**
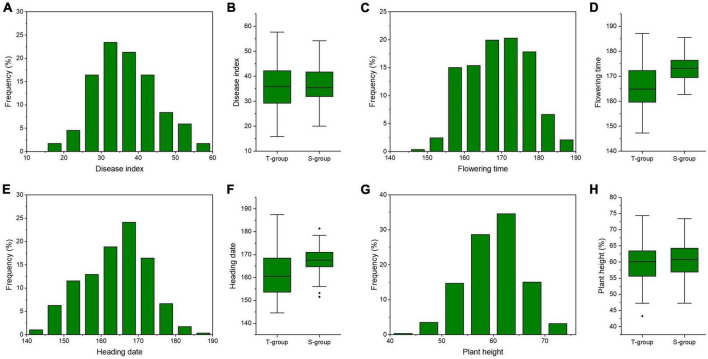
Phenotypic distributions of phenotypic traits among the whole population and subgroups. Frequency distributions of disease index **(A)**, flowering time **(C)**, heading time **(E)**, and plant height **(G)** among 286 *Aegilops tauschii* accessions. Box plots of disease index **(B)**, flowering time **(D)**, heading time **(F)**, and plant height **(H)** among two subgroups. S-group and T-group represent the *Aegilops tauschii* ssp. *strangulata* group and the *Aegilops tauschii* ssp. *tauschii* group, respectively.

In this panel, FCR severity ranged from 0.80 to 2.88, with a mean value of 1.82. The CV of FCR severity was 22.94% (Table 2). The DI values ranged from 15.81 to 57.66, with a mean value of 36.34 (Table 2). Frequency distributions of DI were continuous and normally (Figure 2). The ANOVA revealed significant (*P* < 0.01) genotypic and environmental effects of FCR resistance on the *A. tauschii* natural population. The broad-sense heritability of FCR resistance was 0.65, indicating it was major controlled by genetic effects. Among 286 *A. tauschii*, five accessions had DI lower than 20, indicating these five were moderate resistance to FCR. This five originated from Afghanistan, China, Iran, Turkey, and Uzbekistan (Supplementary Table 1). The five accessions all belonged to the T-group (Supplementary Table 1). The remaining 60 and 221 accessions were moderate and high susceptibility, respectively.

**TABLE 2:** Phenotypic variations and heritability of disease index, flowering time, heading date, and plant height.

Trait	Minimum	Maximum	Mean	SD	CV (%)	Heritability
FCR severity	0.80	2.88	1.82	0.42	22.94	0.65
DI	15.81	57.66	36.34	8.38	23.06	0.65
FT	147.29	187.11	169.07	8.10	4.79	0.82
HD	144.52	187.49	163.79	8.69	5.31	0.81
PH	43.26	74.35	60.05	5.45	9.08	0.54

CV, coefficient of variation; DI, disease index; FT, flowering time; HD, heading date; PH, plant height; SD, standard deviation.

To resolve relationships between FCR resistance and possible agronomic traits, BLUP values across four environments of flowering time (FT), heading date (HD) and plant height (PH) were gathered. The broad-sense heritability of FT, HD, and PH was 0.82, 0.81, and 0.54 (Table 2). It indicated FT and HD were highly inheritable characters, while PH was affected by genetic and environmental effects (Table 2). Frequency distributions of FT, HD, and PH showed continuous and normal distributions (Figure 2). Based on Person’s correlation analysis, correlation coefficients between DI and FT, HD, and PH were 0.12, 0.15, and 0.02, respectively. It indicated that FCR resistance was weakly correlated with FT and HD and not PH.

### Fusarium crown rot resistance loci revealed by genome-wide association study in *Aegilops tauschii* and their associations with flowering time, heading date, and plant height

**FIGURE 3:**
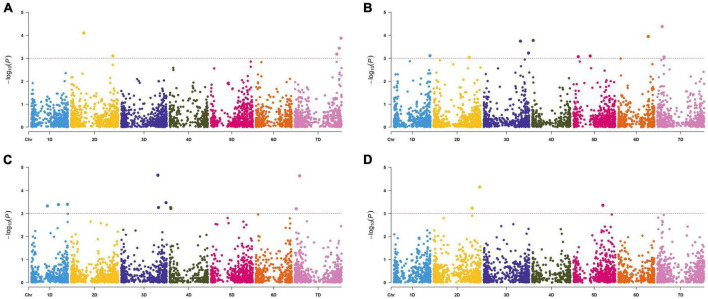
Manhattan plots of marker-trait associations detected by the mixed linear model using 6,739 single nucleotide polymorphisms in 286 *Aegilops tauschii* accessions. Panels **(A–D)** represents marker-trait associations of disease index **(A)**, flowering time **(B)**, heading time **(C)**, and plant height **(D)**. The horizontal dashed line represents the significant threshold –log_10_(*P*) = 3.00.

Based on the threshold value *P* < 0.001 correspondingly −log_10_(*P*) = 3.00, six SNPs were identified for FCR resistance by GWAS in the 286 *A. tauschii* natural population. These loci were located on chromosomes 2D and 7D, explaining 3.90–5.35% phenotypic variations (Figure 3 and Table 3). Among these, two were located on the short arm of chromosome 2D at 177.65 Mb and the long arm of chromosome 2D at 577.73 Mb, respectively. Four SNPs were located on chromosome 7D at 579.21, 612.48, 612.48, and 637.80 Mb, respectively. To resolve relationships between FCR resistance and possible-related agronomic traits, SNPs associated with FT, HD, and PH were also detected by genome-wide association mapping. The results showed that 19 SNPs were significantly associated with FT, HD, and PH (Figure 3 and Table 3). The SNP *GCE8AKX02GFUOC* on chromosome 7D at 67.90 Mb explained the highest phenotypic variation. Eleven SNPs were significantly associated with FT with PVE ranging from 3.80 to 6.90%. These SNPs are located on all seven chromosomes of *A. tauschii*. The SNP *GCE8AKX02GFUOC* on chromosome 7D at 67.90 Mb explained the highest phenotypic variation. For HD, 11 SNPs were identified with PVE ranging from 4.05 to 7.28%. These 11 SNPs were located on chromosomes 1D, 3D, 4D, and 7D, respectively. The SNP G*CE8AKX02GFUOC* associated with HD on chromosome 7D at 67.90 Mb explained the highest phenotypic variation. Three SNPs were significantly associated with PH. Two SNPs were located on chromosome 2D at 531.86 and 637.24 Mb, and one SNP was located on chromosome 5D at 405.67 Mb with PVE ranging from 4.09 to 6.54%. These loci, associated with FT, HD, and PH, were not overlapped loci associated with FCR resistance.

**TABLE 3:** Significant markers associated with disease index, flowering time, heading date, and plant height.

Trait	Marker	Chromosome	Position (Mb)	Allele	MAF	−log_10_(*P*)	PVE (%)
DI	*GB5Y7FA01DPZQ3*	2D	177.65	A/G	0.20	4.10	5.35
DI	*GA8KES401CURCG*	2D	577.73	T/C	0.45	3.10	3.90
DI	*GDRF1KQ02F6UYY*	7D	579.21	A/G	0.47	3.18	4.01
DI	*GBQ4KXB01BSGDP*	7D	612.48	A/G	0.16	3.44	4.39
DI	*GB5Y7FA01B2L7A*	7D	612.48	T/C	0.16	3.44	4.39
DI	*GA8KES402HI5B7*	7D	637.80	T/C	0.36	3.87	5.02
FT	*contig14466*	1D	494.03	T/C	0.49	3.11	3.92
FT	*contig25902*	1D	494.03	A/C	0.49	3.11	3.92
FT	*F5XZDLF02F25YT*	2D	492.27	A/G	0.50	3.03	3.80
FT	*GDS7LZN02FXGIW*	3D	504.23	A/G	0.43	3.75	4.84
FT	*contig10239*	3D	616.14	T/C	0.42	3.22	4.08
FT	*GBB4FNX02JQNSU*	4D	14.34	A/G	0.44	3.77	4.88
FT	*GBF1XID01D8GCW*	5D	67.10	T/G	0.44	3.07	3.85
FT	*GDS7LZN01CBDK1*	5D	231.32	A/G	0.27	3.10	3.90
FT	*contig37461*	6D	412.96	A/G	0.45	3.95	5.13
FT	*GCE8AKX02GFUOC*	7D	67.90	T/C	0.37	4.38	6.90
FT	*F1BEJMU02FHJQ9*	7D	94.52	T/C	0.41	3.05	3.83
HD	*contig54537*	1D	218.89	A/G	0.44	3.33	5.28
HD	*contig30810*	1D	370.94	T/C	0.49	3.39	4.32
HD	*contig14466*	1D	494.03	T/C	0.49	3.40	4.34
HD	*contig25902*	1D	494.03	A/C	0.49	3.40	4.34
HD	*GDS7LZN02FXGIW*	3D	504.23	A/G	0.43	4.66	6.17
HD	*GDEEGVY02JFJRQ*	3D	512.91	T/G	0.47	3.26	4.14
HD	*contig10239*	3D	616.14	T/C	0.42	3.47	4.44
HD	*GBB4FNX02JQNSU*	4D	14.34	A/G	0.44	3.25	4.13
HD	*GA8KES401D0WEU*	4D	16.54	A/C	0.28	3.22	4.07
HD	*GA8KES401ADZCH*	7D	21.62	A/G	0.47	3.20	4.05
HD	*GCE8AKX02GFUOC*	7D	67.90	T/C	0.37	4.63	7.28
PH	*F5XZDLF02IYX94*	2D	531.86	T/C	0.38	3.23	4.09
PH	*F1BEJMU02GJ0B4*	2D	637.24	T/G	0.17	4.15	6.54
PH	*GDEEGVY01BX4MT*	5D	405.67	T/C	0.20	3.36	4.28

DI, disease index; FT, flowering time; HD, heading date; MAF, minor allele frequency; PVE, phenotypic variation explained; PH, plant height.

### Distributions of Fusarium crown rot resistance alleles in T-group and S-group

**FIGURE 4:**
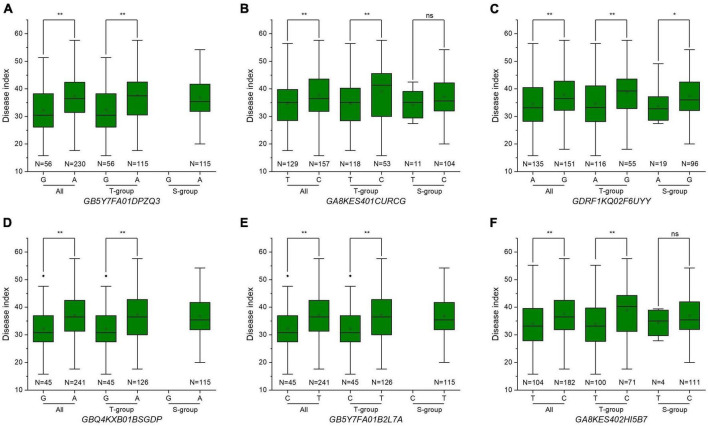
Allele distributions of significant single nucleotide polymorphisms (SNPs) significantly associated with Fusarium crown rot resistance. **(A–F)** Allele distributions of the six significantly associated SNPs *GB5Y7FA01DPZQ3*, *GA8KES401CURCG*, *GDRF1KQ02F6UYY*, *GBQ4KXB01BSGDP*, *GB5Y7FA01B2L7A*, and *GA8KES402HI5B7* among the whole population and subgroups. S-group and T-group represent the *Aegilops tauschii* ssp. *strangulata* group and the *Aegilops tauschii* ssp. *tauschii* group, respectively. *N* represents the number of alleles.

In the whole panel, *A. tauschii* accessions with resistance alleles showed lower DI than accessions with susceptibility alleles (Figure 4 and Table 4), indicating *A. tauschii* accessions with resistance alleles were more resistant to FCR. *A. tauschii* accessions with resistance alleles could decrease by 9.16 to 15.21% DI values, corresponding to increased FCR resistance. Interestingly, most *A. tauschii* accessions with resistance alleles belong to the T-group. Resistance alleles of loci *GB5Y7FA01DPZQ3*, *GBQ4KXB01BSGDP*, and *GB5Y7FA01B2L7A* were only present in T-group accessions (100%) and all absent in S-group accessions (Figures 4A,D,E and Table 4). Resistance alleles were present in 129, 135, and 104 accessions for the other three loci. But only a few accessions with resistance alleles were in S-group, while most were in the T-group. Resistance alleles were only present in 11, 19, and 4 accessions of the S-group but in 118, 116, and 100 accessions of the T-group (Figures 4B,C,F and Table 4). Besides, accessions with 3–6 superior alleles all belonged to the T-group (Supplementary Table 1). These results suggested that *A. tauschii* accessions in the T-group (subspecies *tauschii*) harbored abundant resistance alleles to FCR.

**TABLE 4:** Distributions of superior and inferior alleles for the six significant single nucleotide polymorphisms associated with Fusarium crown rot resistance.

SNP	Allele type		Panel	Allele number		Allele frequency (%)		Phenotype value		Difference (%)	Significance
	Superior	Inferior		Superior	Inferior	Superior	Inferior	Superior	Inferior		
*GB5Y7FA01DPZQ3*	G	A	ALL	56	230	19.58	80.42	32.41 ± 8.22	37.3 ± 8.14	15.09	**
			T-group	56	115	32.75	67.25	32.41 ± 8.22	37.75 ± 9.02	16.48	**
			S-group	0	115	0.00	100.00	–	36.84 ± 7.11	–	–
*GA8KES401CURCG*	T	C	ALL	129	157	45.10	54.90	34.6 ± 8.14	37.77 ± 8.3	9.16	**
			T-group	118	53	69.01	30.99	34.63 ± 8.37	39.07 ± 9.92	12.82	**
			S-group	11	104	9.57	90.43	34.26 ± 4.9	37.11 ± 7.26	8.32	ns
*GDRF1KQ02F6UYY*	A	G	ALL	135	151	47.20	52.80	34.59 ± 8.47	37.9 ± 7.98	9.57	**
			T-group	116	55	67.84	32.16	34.73 ± 8.87	38.7 ± 9.04	11.43	**
			S-group	19	96	16.52	83.48	33.73 ± 5.32	37.45 ± 7.26	11.03	*
*GBQ4KXB01BSGDP*	G	A	ALL	45	241	15.73	84.27	32.21 ± 7.4	37.11 ± 8.32	15.21	**
			T-group	45	126	26.32	73.68	32.21 ± 7.4	37.36 ± 9.28	15.99	**
			S-group	0	115	0.00	100.00	–	36.84 ± 7.11	–	–
*GB5Y7FA01B2L7A*	C	T	ALL	45	241	15.73	84.27	32.21 ± 7.4	37.11 ± 8.32	15.21	**
			T-group	45	126	26.32	73.68	32.21 ± 7.4	37.36 ± 9.28	15.99	**
			S-group	0	115	0.00	100.00	–	36.84 ± 7.11	–	–
*GA8KES402HI5B7*	T	C	ALL	104	182	36.36	63.64	33.98 ± 8.34	37.69 ± 8.1	10.92	**
			T-group	100	71	58.48	41.52	33.97 ± 8.45	38.88 ± 9.25	14.45	**
			S-group	4	111	3.48	96.52	34.31 ± 4.82	36.93 ± 7.17	7.64	ns

**Significant at *P* < 0.01; ns, not significant; “–” data unavailable.

**FIGURE 5:**
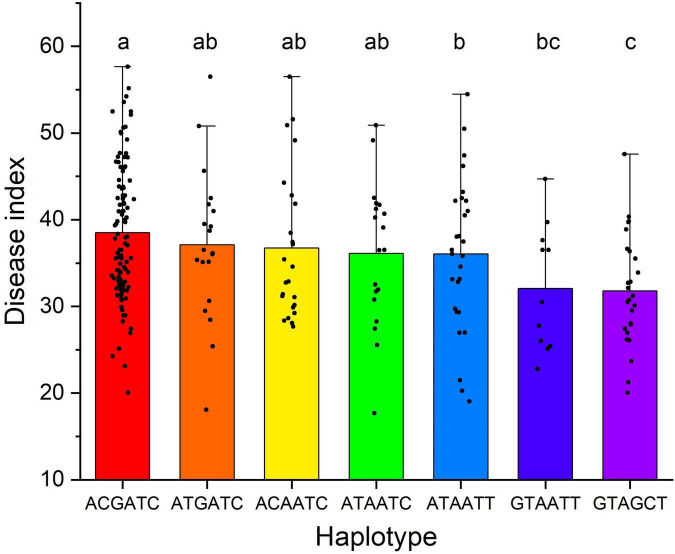
Haplotype analysis based on single nucleotide polymorphisms significantly associated with Fusarium crown rot (FCR) resistance. The different lowercase letters between haplotypes denote FCR resistance were significant (*P* < 0.05) differences. The seven haplotypes from left to right contained 0, 1, 1, 2, 3, 4, and 6 resistance alleles and presented 107, 20, 24, 19, 28, 11, and 26 accessions, respectively.

Haplotypes based on these six SNP were also analyzed to detect the additive effect. Seven haplotypes that included more than ten accessions were used to compare the difference between groups. It showed that accessions with six superior alleles (the GTAGCT haplotype) were significantly (*P* < 0.05) more resistant than those with 0–3 superior alleles (Figure 5 and Supplementary Table 2). Accessions with 3–4 superior alleles were significantly (*P* < 0.05) more resistant than those without any superior allele. It indicated that pyramiding superior alleles could effectively increase FCR resistance of *A. tauschii* accessions.

### Prediction of candidate genes associated with Fusarium crown rot resistance

The *A. tauschii* genome reference sequence (Aet 4.0) was used to identify the candidate genes possibly associated with the significant FCR resistance loci. Seventy-three HC genes were around the six significant SNP for FCR resistance (Supplementary Table 3). The functions of HC genes were annotated using KOBAS 3.0. Fifty-six HC genes were successfully annotated, and 22 were homologous to the known function genes in arabidopsis. Six were selected as candidate genes based on the gene annotation information (Table 5). The first gene, *AET2Gv20439300*, was around at significant SNP *GB5Y7FA01DPZQ3*. It encoded protein tyrosine phosphatase 1 and was homologous to *AtPTP1* (Xu et al., 1998). *AET2Gv21053400* encoding lipid transfer protein 4 was considered the candidate gene for the locus on the long arm of chromosome 2D. It was homologous to the arabidopsis gene *AtLTP4*, a member of the pathogenesis-related (PR) protein family (Backer et al., 2019). Candidate genes *AET7Gv21153400*, *AET7Gv21254300*, and *AET7Gv21255000*, included BTB-POZ and MATH domain and were homologous to *AtBPM6*, *AtBPM1*, and *AtBPM2*, respectively (Weber et al., 2005). The last gene, *AET7Gv21339800*, could encode eukaryotic aspartyl protease family protein.

**TABLE 5:** Candidate genes associated with Fusarium crown rot resistance.

*Aegilops tauschii* gene	Significant marker	Arabidopsis gene	Known gene name in Arabidopsis	Gene description	References
*AET2Gv20439300*	*GB5Y7FA01DPZQ3*	*AT1G71860*	*AtPTP1*	Protein tyrosine phosphatase 1	Xu et al., 1998
*AET2Gv21053400*	*GA8KES401CURCG*	*AT5G59310*	*AtLTP4*	Lipid transfer protein 4	Backer et al., 2019
*AET7Gv21153400*	*GDRF1KQ02F6UYY*	*AT3G43700*	*AtBPM6*	BTB-POZ and MATH domain 6	Weber et al., 2005
*AET7Gv21254300*	*GBQ4KXB01BSGDP, GB5Y7FA01B2L7A*	*AT5G19000*	*AtBPM1*	BTB-POZ and MATH domain 1	Weber et al., 2005
*AET7Gv21255000*	*GBQ4KXB01BSGDP, GB5Y7FA01B2L7A*	*AT3G06190*	*AtBPM2*	BTB-POZ and MATH domain 2	Weber et al., 2005
*AET7Gv21339800*	*GA8KES402HI5B7*	*AT1G49050*		Eukaryotic aspartyl protease family protein	

## Discussion

### Fusarium crown rot resistance alleles is abundant in *Aegilops tauschii* ssp. *tauschii* but rarely in *Aegilops tauschii* ssp. *strangulata*

*Aegilops tauschii*, the diploid wild progenitor of the D-genome of common wheat, is a reservoir of genetic diversity for improving bread wheat biotic and abiotic resistance/tolerance. Previous studies have identified resistance/tolerance genes/loci response to phosphorus deficiency, drought, stripe rust, and powdery mildew resistance (Liu et al., 2015a; Qin et al., 2016; Zhang et al., 2019b; Xue et al., 2022). It has proved that superior genes identified from *A. tauschii* could be quickly and efficiently used to improve hexaploid wheat (Zhang et al., 2019b; Awan et al., 2022; Gaurav et al., 2022). For example, the stripe rust resistance allele *YrAS2388R* on chromosome 4DS was cloned from *A. tauschii*. It was only present in *A. tauschii* (100 and 19% present in subspecies *strangulata* and *tauschii*, respectively) and the *A. tauschii* -derived synthetic wheat and absent in 100% of 461 tested common wheat lines (Zhang et al., 2019b). The synthetic hexaploid wheat method and transgenic test have proved that the allele *YrAS2388R* can be transferred into hexaploid wheat and improve the resistance to stripe rust (Zhang et al., 2019b). Recently, the first wheat cultivar Shumai1675 carrying *YrAS2388R* with high resistance to stripe rust was released in China. We identified five moderate resistance accessions and six SNPs associated with FCR resistance in 286 worldwide *A. tauschii* accessions in the present study. Interestingly, these five accessions with moderate resistance originated from five countries (Afghanistan, China, Iran, Turkey, and Uzbekistan), but all belonged to the subspecies *tauschii* (Supplementary Table 1). These results suggested subspecies *tauschii* may be richer in genetic resources of FCR resistance than subspecies *strangulata.* It was also found that resistance alleles were primarily present in the T-group and rarely present in S-group (Table 4). For three loci, resistance alleles were only present in accession of the T-group and 100% absent in accessions of the S-group. For the rest three, only 11, 19, and 4 resistance alleles were from accessions of the S-group. Besides, *A. tauschii* ssp. *strangulata* was known to be the D-genome donor of wheat. A study has proven that D-genome consensus carried only 68 (2.7%) alleles from subspecies *tauschii* and 2,406 (97.3%) alleles from subspecies *strangulata* (Singh et al., 2019). It indicated that subspecies *tauschii* contains more private alleles that were not transferred into wheat than subspecies *strangulata*. These results suggested that *A. tauschii* ssp. *tauschii* is the focus material for mining FCR resistance gene/loci in our further work.

### Loci associated with Fusarium crown rot resistance revealed by genome-wide association study in the *Aegilops tauschii* natural population

QTL for FCR resistance have been identified in barley and wheat. To date, QTL of FCR resistance have been detected on chromosomes 1H, 2H, 3H, 4H, 5H, and 6H in barley (Chen et al., 2013; Liu C. et al., 2015; Gao et al., 2019). For example, two QTL conferring FCR resistance were detected on chromosomes 1H and 3H using a recombinant inbred line population derived from Baudin and AWCS079 (Chen et al., 2013). Using another recombinant inbred line population derived from Fleet and AWCS799, QTL were detected on chromosomes 1H, 2H, 3H, 4H, 5H, and 6H, but only *Qcrs.caf-6H* was detected in all four experiments (Gao et al., 2019). In wheat, QTL for FCR resistance have been detected on chromosomes 1A, 1B, 1D, 2A, 2B, 2D, 3A, 3B, 3D, 4B, 4D, 5D, 6A, 6B, and 6D (Liu C. et al., 2015; Su et al., 2021; Yang et al., 2021). These QTL are distributed on all D group chromosomes except chromosome 7D. Using one recombinant inbred line population derived from EGA Wylie and Sumai3, a major locus *Qcrs.cpi-5D* was detected in explained up to 31.1% of the phenotypic variance (Zheng et al., 2014). Besides, a major and stable QTL *Qcrs.cpi-2D* was detected in three replicated trials with PVE up to 20.2%. The simple repeat sequence (SSR) marker *cfd73* linked closely to *Qcrs.cpi-2D* was used to genotype in two additional recombinant inbred line populations, indicating that *Qcrs.cpi-2D* could increase FCR resistance (Zheng et al., 2014). Based on these closely linked markers, *Qcrs.cpi-2D* was mapped on the long arm of chromosome 2D at 556.62–602.73 Mb of the Chinese Spring Reference Sequence physical map v2.1 (IWGSC RefSeq v2.1). In the present study, the SNP *GA8KES401CURCG* on the long arm of chromosome 2D was significantly associated with FCR resistance. Based on the SNP flanking sequence, *GA8KES401CURCG* was mapped on chromosome 2D at 582.53 Mb, overlapped with the genomic region of *Qcrs.cpi-2D.* These results indicated that these two loci might be the same. The resistance allele of this locus was present in both the T-group and S-group. It is well known that *A. tauschii* ssp. *strangulata* was the D-genome donor (Singh et al., 2019). Thus, this locus may be originated from *A. tauschii* and transferred into hexaploid wheat due to the natural hybridization of tetraploid wheat and *A. tauschii* about 8,000–10,000 years ago. However, more evidence is needed to prove it. In another previous study, the QTL flanking with SSR markers *gwm484* and *gwm102* on the short arm of chromosome 2D was significantly associated with FCR in field trials in two doubled haploid populations (Martin et al., 2015). This QTL was located on chromosome 2D at 50.63 Mb. In the present study, an SNP *GB5Y7FA01DPZQ3* was significantly associated with FCR resistance on the short arm of chromosome 2D at 178.40 Mb. These two loci were far from each other. Thus, the locus identified in this study may be novel. Besides, four SNPs were significantly associated with FCR resistance on the long arm of chromosome 7D. Before this study, no loci/gene for FCR resistance was identified on chromosome 7D in *A. tauschii* or wheat. These results showed that *A. tauschii* is a valuable source of novel FCR resistance loci for wheat improvement.

### Fusarium crown rot resistance loci are not associated with flowering time, heading date, and plant height in *Aegilops tauschii*

Heading date and PH are crucial agronomic traits in plants that determine many crop productivity. Previous studies have found that PH and HD affected FCR resistance in wheat (Liu et al., 2010; Liu C. et al., 2015; Zheng et al., 2014). Co-located QTL for resistance and HD and PH, and these loci impeded developing closely linked markers associated with FCR resistance (Liu C. et al., 2015). The present study also analyzed to detect possible relationships between FCR resistance and traits of agronomic importance in *A. tauschii*, FT, HD, and PH. In the present study, FCR resistance was weakly correlated with FT and HD and was not correlated with PH. A genome-wide association analysis was performed for these three traits to understand their relationship further. Eleven, 11, and 3 SNP were significantly associated with FT, HD, and PH, respectively (Table 4). The results showed no loci between FCR resistance and FT, HD, and PH were overlapped. It indicated that FCR resistance was independent of FT, HD, and PH in the *A. tauschii* natural population. Thus, these loci that did not influence these possible plant development traits are better used in breeding programs.

### Potential candidate genes associated with Fusarium crown rot resistance loci

Using GWAS in a wheat population, the FCR resistance gene *TaDIR-B1* encoding dirigent protein has been found and validated. In the present study, six candidate genes were identified for these candidate regions and will further validate their function response to FCR (Table 5). The SNP *GB5Y7FA01DPZQ3* on chromosome 2D was significantly associated with FCR resistance. *AET2Gv20439300* is flanking this locus and could encode protein tyrosine phosphatase 1. It was homologous to *AtPTP1* (Xu et al., 1998). *AtPTP1* is a member of phosphotyrosine-specific protein phosphatases in arabidopsis (Xu et al., 1998). The expression of *AtPTP1* was altered in response to different environmental stresses. It was also confirmed that *AtPTP1* repressed salicylic acid biosynthesis in the autoimmune-like response caused by toll interleukin one receptor/nucleotide-binding/leucine-rich repeat (*TIR-NB-LRR*) receptor-like resistance gene (Bartels et al., 2009). The SNP *GB5Y7FA01DPZQ3* was significantly associated with FCR resistance and located on the long arm of chromosome 2D. *AET2Gv21053400* could encode lipid transfer protein four and be homologous to *AtLTP4*. *AtLTP4* is a member of the PR protein family, which plays a fundamental role in a plant’s response to pathogen challenges (Backer et al., 2019). In wheat, *Fusarium* species infection caused the induction of several PR proteins during the development of seedlings (Caruso et al., 1999). In garlic (*Allium sativum* L.), it was also found that PR genes, which encoded *AsPR1*, *AsPR2*, *AsPR4*, and *AsPR5* proteins, could mediate protection against some *Fusarium* species infection (Anisimova et al., 2021). Thus, *AET2Gv21053400* was suggested as a candidate gene for the locus on the long arm of chromosome 2D. Besides, three genes, *AET7Gv21153400*, *AET7Gv21254300*, and *AET7Gv21255000*, contained BTB-POZ and MATH domains and were homologous to *AtBPM6*, *AtBPM1*, and *AtBPM2*, respectively (Weber et al., 2005). Increasing evidence has found that BTB-POZ and MATH protein family plays a vital role in plant defense regulation (Xu et al., 2003; Zavaliev et al., 2020). In soybean, *GmBTB/POZ* encoding BTB/POZ domain-containing nuclear protein could positively regulate the response to fungal infection (Zhang et al., 2019a). It was also reported that *NbBTB* encoding a BTB/POZ domain-containing protein could regulate plant immunity in *Nicotiana benthamiana* (Zhao et al., 2022). Besides, MATH domain protein is associated with resistance to fungal disease in arabidopsis (Cosson et al., 2010) and apricot (*Prunus armeniaca* L.) (Mariette et al., 2016). The predicted gene *Sobic.010G222400* containing BTB/POZ and MATH domains, was identified as a candidate for resistance to sorghum grain mold caused by *Fusarium* species (Cuevas et al., 2019). Thus, *AET7Gv21153400, AET7Gv21254300*, and *AET7Gv21255000* may regulate FCR resistance in *A. tauschii*. The last candidate gene was *AET7Gv21339800*, which could encode an aspartyl protease family protein. The aspartyl protease plays a vital role in plant-pathogen interactions (Figueiredo et al., 2021). It has been demonstrated that *AtAPCB1* encoding aspartyl protease is necessary for fungal resistance in Bcl-2-associated athanogene processing (Li et al., 2016). *OsAP47* encoding aspartyl protease is associated with resistance to black-streaked dwarf virus disease and southern rice black-streaked dwarf virus disease (Wang et al., 2022). These six candidate genes were identified for FCR resistance, and further analysis, such as qRT-PCR and the transgenic test, is needed to confirm the function.

## Data availability statement

The original contributions presented in this study are included in the article/Supplementary material, further inquiries can be directed to the corresponding author.

## Author contributions

YuL and QW drafted and revised the manuscript and contributed to data analysis. HC and NY performed the phenotypic evaluation and helped with data analysis. FW, ZW, and CL performed part of the statistical analyses. YaL designed and coordinated the study and revised the manuscript. All authors have read and approved the final manuscript for publication.

## Supplementary material

Supplementary Figure 1 Distributions of 6,739 single nucleotide polymorphisms on *Aegilops tauschii* chromosomes. Color represents the number of single nucleotide polymorphisms within a 1 Mb window size, from 0 (white) to 10 (red).

Supplementary Figure 2 Delta *K* over ten repetitions estimated by population structure analysis.

Supplementary Table 1 Detail information and phenotypic data of 286 *Aegilops tauschii* accessions used in this study.

Supplementary Table 2 Haplotype analysis based on single nucleotide polymorphisms significantly associated with Fusarium crown rot (FCR) resistance.

Supplementary Table 3 Detail information of predicted genes flanking significant markers associated with Fusarium crown rot resistance.
