# Supplementary figures and images for: Genome-wide association mapping of Fusarium crown rot resistance in Aegilops tauschii
Source: Front Plant Sci. 2022 Sep 30;13:998622. doi: 10.3389/fpls.2022.998622 (PMC9562832; doi:10.3389/fpls.2022.998622)

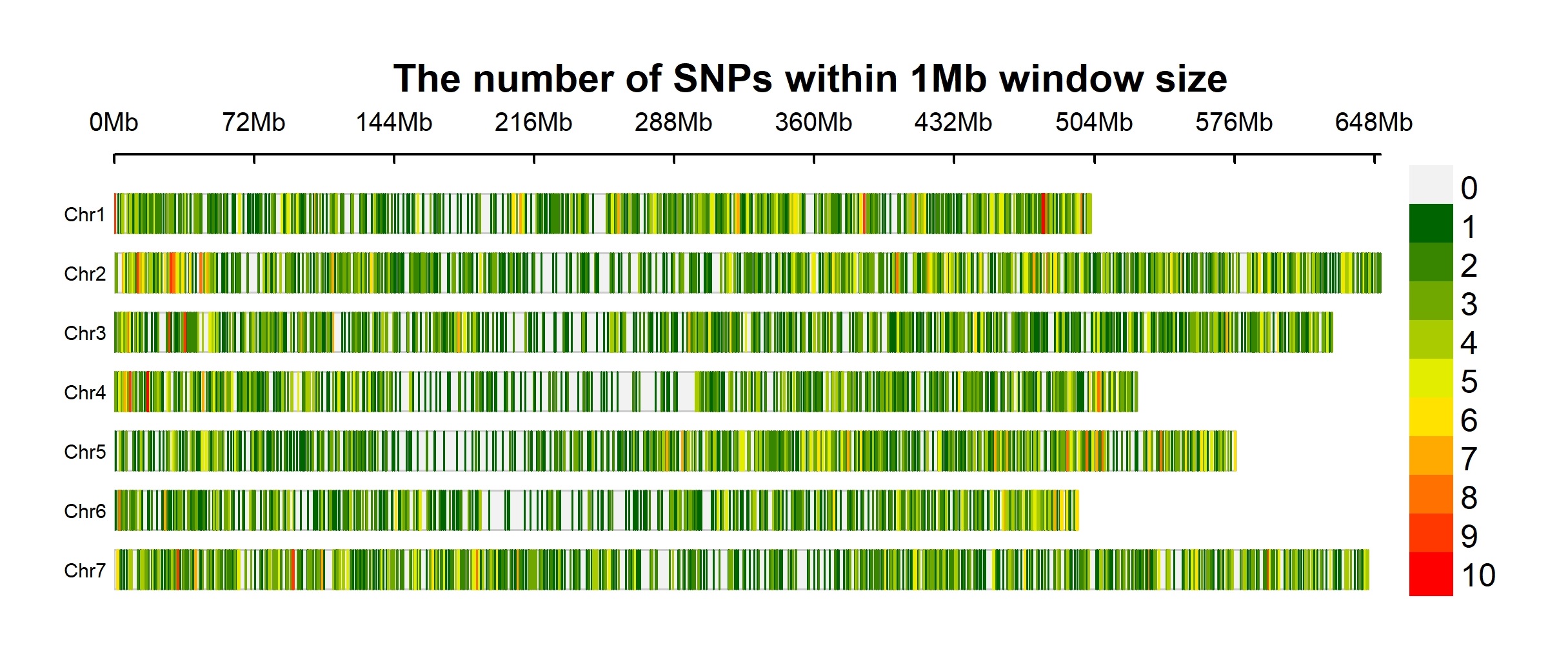

Supplement: Supplementary Figure 1 — Distributions of 6,739 single nucleotide polymorphisms on Aegilops tauschii chromosomes. Color represents the number of single nucleotide polymorphisms within a 1 Mb window size, from 0 (white) to 10 (red). [file Image_1.JPEG]

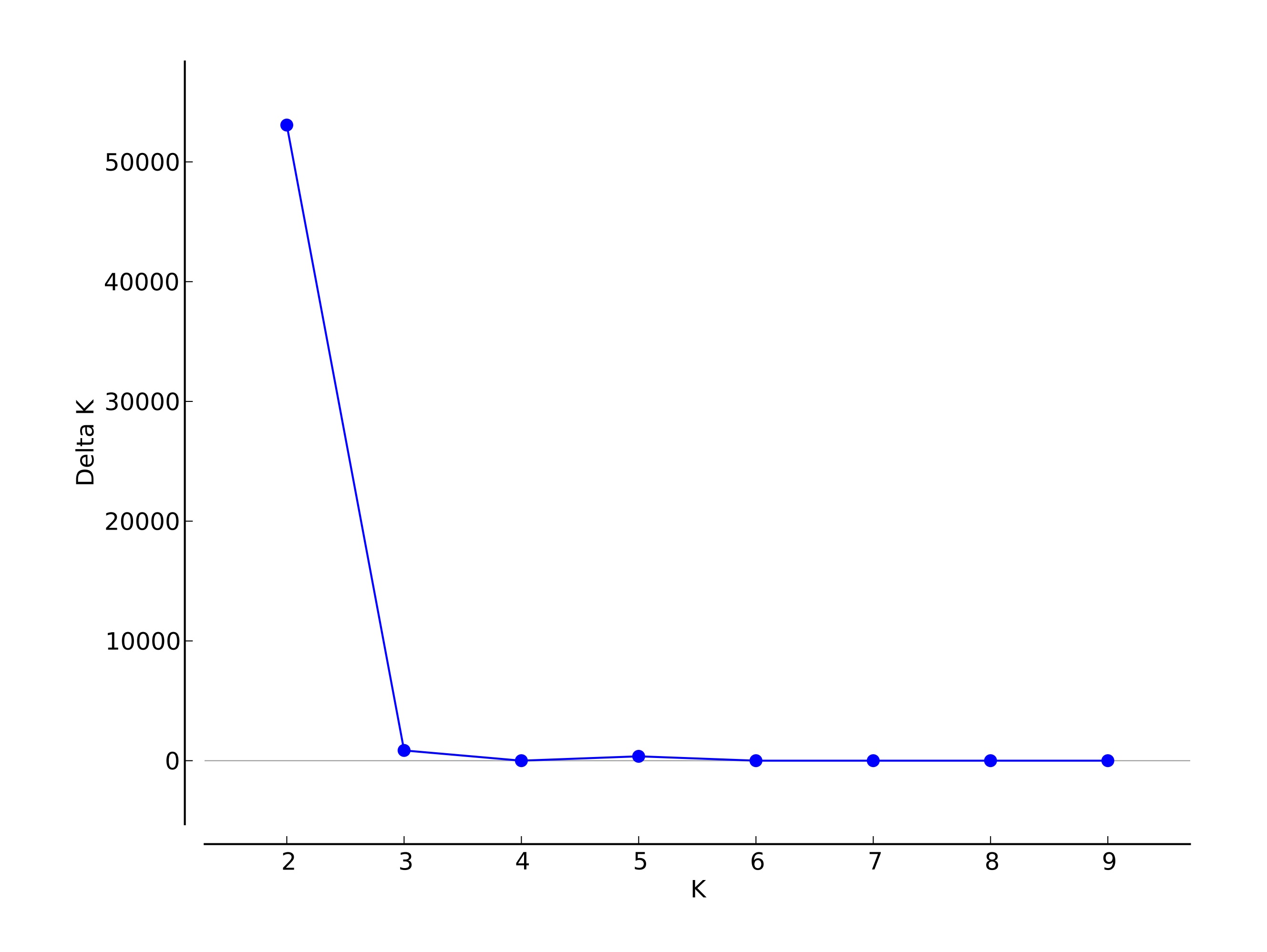

Supplement: Supplementary Figure 2 — Delta K over ten repetitions estimated by population structure analysis. [file Image_2.JPEG]
